# Musculoskeletal Diseases: From Molecular Basis to Therapy

**DOI:** 10.3390/biomedicines12010032

**Published:** 2023-12-22

**Authors:** Elisa Belluzzi, Assunta Pozzuoli, Pietro Ruggieri

**Affiliations:** 1 Musculoskeletal Pathology and Oncology Laboratory, Department of Surgery, Oncology and Gastroenterology DiSCOG, University of Padova, Via Giustiniani 3, 35128 Padova, Italy; elisa.belluzzi@unipd.it; 2 Orthopedics and Orthopedic Oncology, Department of Surgery, Oncology and Gastroenterology DiSCOG, University of Padova, Via Giustiniani 3, 35128 Padova, Italy; 3 Centre for Mechanics of Biological Materials, University of Padova, 35131 Padova, Italy

Musculoskeletal diseases (MSDs) comprise a plethora of different disorders (more than 150 conditions) affecting the locomotor system. Importantly, they are associated with significant morbidity and disability, impacting the quality of life of patients. The most common MSDs are osteoarthritis (OA), low-back pain (LBP), neck pain (NP), rheumatoid arthritis (RA), and gout [1].

A recent analysis of the Global Burden of Disease published in 2022 estimated that approximately 1.71 billion people globally are affected by musculoskeletal disorders [2]. Although MSDs are widespread and the number of affected individuals is expected to increase as the population ages, MSD research has received little attention, likely because MSDs are rarely fatal and are assumed to be irreversible pathologies [1,2]. Thus, a better understanding of the etiology, biomarkers, as well as new and more effective therapeutic treatments, are needed. In this context, the purpose of this Special Issue, entitled “Musculoskeletal Diseases: From Molecular Basis to Therapy”, is to report on advances in pathophysiological mechanisms, the identification of biomarkers, and preclinical and clinical therapeutic approaches to MSDs. This Special Issue includes three narrative reviews [3,4,5], two systematic reviews [6,7], and nine original research articles [8,9,10,11,12,13,14,15,16] on different topics about MSDs.

Bone and muscle have a close cross-talk and are considered a single functional system [17]. While the role of the parathyroid hormone (PTH) in calcium and phosphate homeostasis is well known, in bone metabolism [18], little is known about its effects on skeletal muscle. Romagnoli et al. investigated the effects of PTH on human muscle, showing that stimulation of 30 min of PTH can affect the early stage of human satellite cells by increasing the expression of myogenic markers and promoting the myogenic differentiation process [10].

Kumar et al. wrote an interesting update on the role of metalloproteinases (MMPs) in MSDs [4]. In particular, they discussed the involvement of different MMPs in skeletal muscle regulation, muscle injury and repair, inflammation and oxidative stress, and muscular dystrophy.

Skeletal muscle pathologies seem to have a strong correlation with reactive oxygen species (ROS) as oxidative damage increases the aging process, and it is associated with the development of various diseases [19]. Superoxide dismutases (SODs) are a group of three metalloenzymes that catalyze the dismutation of superoxide radicals to O_2_ and H_2_O_2_ [20]. Shibuya et al. used a muscle-specific SOD2-deficient mouse (muscle-Sod2^−/−^), a model of muscle fatigue, to investigate the effects of 11 compounds (polyphenols, vitamins, and amino acids) on treadmill exercise [13]. They showed that gossypin, genistein, kaempferol, taxifolin, fumaric acid, β-hydroxy-β-methylbutyrate, tempol, astaxanthin, MnTE-2-PyP, troglitazone, and Trolox have positive effects on treadmill exercise, while citric acid, phosphocreatine, and nicotinamide have negative effects. These results suggest that functional food intake with antioxidant activity could improve motor function, which deserves to be explored [13].

Several MSDs are characterized by a bone mineral density (BMD) decrease causing bone fragility. Among these pathologies, testosterone deficiency syndrome, or hypogonadism, is a multi-factorial condition in which there is low production of testosterone in males, resulting in a reduction in BMD and muscle mass [21]. The primary treatment is testosterone replacement therapy. However, various research groups are exploring the use of nonpharmacological interventions such as exercise. In this regard, Stratos et al. investigated the effects of distinct training modalities (uphill, downhill, and level running) on bone and muscle in hypogonadal male (castrated) rats compared to sham rats [9]. Castration induced a lower bending stiffness and a decreased trabecular BMD of the tibia at 8 and 12 weeks, which were restored in castrated rats undergoing downhill and uphill running. However, soleus muscle force measurements revealed decreased tetanic force in castrated rats at week 12, while uphill and downhill training restored force to sham group levels and led to muscle hypertrophy compared to castrated animals. Interestingly, treadmill training was able to counteract the decrease in muscle fiber diameter induced by orchiectomy. Therefore, treadmill training seems to improve muscle regeneration and mitigate osteoporosis in this model [9].

Bone fragility is also present in Osteogenesis Imperfecta (OI), a genetic disorder with a high bone turnover rate due to an altered activity of both osteoclasts and osteoblasts. The best treatment of OI in children is represented by biphosphonates (BPs) that suppress osteoclast activity and survival [22]. However, BP treatment increases creatine kinase (CK), an enzyme involved in cellular energetic metabolism, but the exact mechanism is still unclear. Therefore, Faienza et al. evaluated the correlation between osteoclastogenesis and the isoenzyme brain-type creatine kinase (CK-BB) released from mature osteoclasts of patients with OI vs. healthy donors [11]. Osteoclasts were cultured with neridronate, a BP currently used for OI treatment, in the presence or absence of the pro-osteoclastogenic cytokines macrophage colony-stimulating factor (MCSF) and receptor activator of nuclear kappa-B ligand (RANKL). An increase in CK-BB levels in the medium of OI and healthy osteoclasts cultured with 3 µmol/L neridronate was found, and its levels increased in the presence of MCSF and RANKL. On the contrary, the exposure to lower concentrations of neridronate determined a significant decrease in CK-BB release in both study groups. The authors showed that neridronate treatment was associated with a decrease (dose-dependent) in tartrate-resistant acid phosphatase (TRAP)+ osteoclasts in both groups associated with activating the apoptotic process. This result supports the idea that CK-BB levels increase in the serum of children affected by OI in treatment with BPs could be secondary to its secretion by osteoclasts undergoing apoptosis [11].

BMD decrease, associated with an alteration of bone microstructure, is also present in osteoporosis, one of the most common chronic MSDs of elderly people that leads to an increase in skeletal fragility and fractures [23]. Dual-energy X-ray absorptiometry (DEXA) is the gold standard for measuring BMD but has high costs, and it is a 2-dimensional technique. Quantitative Bone Ultrasound (QUS) is a non-invasive, economical, portable tool and does not use ionizing radiations that can be used to evaluate osteoporosis and fracture risk [24]. Escobio-Prieto et al. assessed the use of DEXA vs. calcaneal QUS in a systematic review to determine and analyze the use of the latter in terms of bone disease prevention and diagnosis. They concluded that the calcaneal QUS tool may be a promising method to evaluate BMD in elderly people, facilitating its prevention and diagnosis [7].

Osteoporosis can also coexist with sarcopenia in so-called osteosarcopenia, which is now considered as a new multifactorial syndrome affecting elderly people [25,26]. Interestingly, pain is significantly associated with osteoporosis and sarcopenia. Bonanni et al. conducted a narrative review with the aim of summarizing the current knowledge on the molecular mechanisms involved in the pain development in osteosarcopenia and the potential countermeasures to be taken [3]. Today, there is no specific therapy for osteosarcopenia as well as for associated pain. Thus, the review highlighted the importance of developing strategies and therapies to counteract musculoskeletal pain in these patients other than preserving musculoskeletal structure/function and slowing down the progression of the syndrome [3].

Sarcopenia is a disease characterized by a progressive and generalized loss of muscle mass and function [27]. While it is widely accepted that physical training should be a fundamental component of sarcopenia treatment [28], the role of nutritional supplementation and the use of a pharmaceutical approach is still controversial. Mellen et al. performed an interesting systematic review to investigate the impact of nutritional and drug approaches on the health and quality of life of sarcopenic patients [6]. The review reported that creatine, leucine, branched-chain amino acids, omega 3, and vitamin D can show benefits. Regarding the pharmacological approach, metformin, statin, glucagon-like peptide-1 receptor agonists, losartan, growth hormone, and dipeptidyl peptidase 4 inhibitors can alter the metabolic parameters of sarcopenic patients. However, there is still a need to develop new clinical trials in order to evaluate the effects of nutrition and pharmacological therapies on patients with different types/stages of sarcopenia to assess the roles and outcomes of interventions [6].

OA, as reported before, is one of the five most common MSDs and can involve any joint but typically affects the hand, the knee, and the hip. It is a complex and multi-factor (gender, genetics, obesity, metabolic diseases, age, and previous injury) disease involving all joint tissues [29]. One of the most affected tissues in OA is cartilage, which exhibits a change in its biomechanical behavior [30]. Chondrocytes, which are the only cell type present in cartilage, undergo several changes in OA. The chondrocyte embedded in the pericellular matrix is called a chondron. The review of Pettenuzzo et al. describes both chondrocyte and chondron biomechanics in healthy and OA conditions, also reporting a critical perspective on different features that could affect the measurements [5]. Moreover, the review pointed out the need to report in detail the methods used in studies evaluating the biomechanical behavior of cells (i.e., method of cell isolation and storage) [5].

OA is a burden that impacts not only humans but also animals, particularly dogs [31]. In 2016, grapiprant, an E prostanoid 4 (EP4) receptor antagonist that exhibits significant anti-inflammatory effects, was approved by the FDA Veterinary Medicine Center to control pain and inflammation associated with osteoarthritis in dogs. Gumulka et al. evaluated the influence of external factors (i.e., pH, temperature, and incubation time) on the stability of this active substance, taking into account the storage and use conditions, which directly impact the safety of its use. The obtained results allowed us to indicate also potential pathways of grapiprant degradation [15].

Another major MSD is LBP, which determines great cost for society [32]. Intervertebral disc degeneration (IDD) is one of the major causes of LBP [33]. IDD is characterized by an increase in inflammatory cytokines in the intervertebral disc, and it has been demonstrated that macrophages (Mφs) are able to infiltrate the nucleus pulposus (NP) after the rupture of the annulus fibrosus in the early stage of IDD [34]. Dou et al. investigated the effects of melatonin, a hormone produced mainly by the pineal gland and involved in regulating circadian rhythms, on macrophage polarization in IDD [8]. Melatonin ameliorated NP cell injury caused by inflammation in vitro and vivo, partially inhibiting M1-type polarization of Mφ by regulating the SIRT1/Notch signaling pathway. Therefore, melatonin treatment could represent a promising therapeutic method for the management of IDD.

Among non-invasive treatments, physiotherapy and biophysical stimulation play an important role in the treatment of several MSDs affecting different tissues (i.e., bones and connective tissues), including joints [35]. Crăciun et al. reported the results of a non-randomized clinical trial of patients affected by temporomandibular dysfunctions with cervical spine involvement [14]. The effectiveness of physiotherapeutic treatments applied to temporomandibular and cervical areas was evaluated in the case of an existing condition in one of the two regions. Patients underwent either conservative drug and physiotherapy treatments or only drug treatment over a period of 3 months. This study showed the efficacy of physiotherapeutic treatments of both regions with the reduction in symptoms and maintenance of the functional state, thus improving the quality of life of the patients [14].

Another non-invasive procedure is represented by biophysical stimulation that could be effectively applied in the treatment of delayed fracture healing in order to enhance bone regeneration [36]. De Francesco et al. reported the use of biophysical stimulation (pulsed electromagnetic fields and capacitive coupling electric fields) in patients who underwent surgical fixation of hand phalanx with delayed union at 30 days from surgery [16]. Two groups of patients treated either with surgery (control group) or surgery associated with biophysical stimulation were compared using digital radiograms. The index of relative bone healing and the total active motion were calculated each month after surgery starting from 30 days and followed up for a total of 6 months after stimulation. An improvement in fracture healing was obtained in patients treated with biophysical stimulation in terms of radiographic quality, bone repair, and relative bone density index when compared with the control group. Therefore, an early administration of biophysical stimulation supports fracture healing and improves the union rate, thus reducing immobilization time and envisaging an early rehabilitation interval with better outcomes [16].

Hands could also be affected by several diseases involving palmar connective tissues. Among these, carpal tunnel syndrome (CTS) and Dupuytren’s disease (DD) represent two diseases associated with tissue fibrosis, whose pathophysiology needs to be better elucidated. Tripković et al. analyzed the proliferation and expression of several profibrotic factors, including fibroblast growth factors (FGFs), connective tissue growth factor (CTGF), in blood vessel walls and surrounding connective tissue of CTS and DD patients (flexor retinaculum and fibrotic cords and adjacent unaffected palmar fascia, respectively); healthy palmar fascia from CTS patients were used as control samples [12]. Increased proliferation and expression of FGFR1, FGFR2, and CTGF were found in the blood vessel walls and connective tissue cells of both affected and unaffected DD samples, indicating that molecular changes also occur in the apparently healthy unaffected palmar fascia. Therefore, a more extensive excision of palmar fascia is advised during surgical treatment of DD. Conversely, minor changes in profibrotic factors were found in the flexor retinaculum of CTS patients, suggesting a lesser involvement in this disease. Also, a modified expression of TGF-1 and syndecan-1 in DD-associated sweat glands was found, suggesting their possible role in the pathophysiology of the disease [12].
